# Aggrecan is required for chondrocyte differentiation in ATDC5 chondroprogenitor cells

**DOI:** 10.1371/journal.pone.0218399

**Published:** 2019-06-17

**Authors:** Juanita K. Hodax, Jose Bernardo Quintos, Philip A. Gruppuso, Qian Chen, Salomi Desai, Chathuraka T. Jayasuriya

**Affiliations:** 1 Department of Pediatrics, Division of Pediatric Endocrinology, The Warren Alpert Medical School of Brown University and Hasbro Children’s Hospital, Providence, RI, United States of America; 2 Department of Orthopedics, The Warren Alpert Medical School of Brown University, Providence, RI, United States of America; Mayo Clinic Minnesota, UNITED STATES

## Abstract

Aggrecan is an integral component of the extracellular matrix in cartilaginous tissues, including the growth plate. Heterozygous defects in the aggrecan gene have been identified as a cause of autosomal dominant short stature, bone age acceleration, and premature growth cessation. The mechanisms accounting for this phenotype remain unknown. We used ATDC5 cells, an established model of chondrogenesis, to evaluate the effects of aggrecan deficiency. ATDC5 aggrecan knockdown cell lines (AggKD) were generated using lentiviral shRNA transduction particles. Cells were stimulated with insulin/transferrin/selenium for up to 21 days to induce chondrogenesis. Control ATDC5 cells showed induction of *Col2a1* starting at day 8 and induction of *Col10a1* starting at day 12. AggKD cells had significantly reduced expression of *Col2a1* and *Col10a1* (p<0.0001) with only minimal increases in expression over time, indicating that chondrogenesis was markedly impaired. The induction of *Col2a1* and *Col10a1* was not rescued by culturing of AggKD cells in wells pre-conditioned with ATDC5 extracellular matrix or in co-culture with wild-type ATDC5 cells. We interpret our studies as indicating that aggrecan has an integral role in chondrogenesis that may be mediated through intracellular mechanisms.

## Introduction

Aggrecan is a core protein with chondroitin and keratan sulfate side chains that is an integral part of the extracellular matrix in cartilaginous tissue [1]. Heterozygous defects in the aggrecan gene have been identified as a cause of autosomal dominant short stature, bone age acceleration, and premature growth cessation, all of which are consistent with a role for aggrecan in the cartilaginous growth plate. Of the 20 families thus far identified with so-called “aggrecanopathies” [2,3], 12 of the families also have early onset osteoarthritis, and 11 have degenerative intervertebral disc disease [3–5]. Of particular significance to the phenotype in these families is bone age acceleration, an atypical finding in short stature of other etiologies. Having identified a family with a defect in the aggrecan gene [5], we undertook studies to elucidate the mechanism by which aggrecan mutations lead to impaired long bone growth in concert with bone age acceleration. Our hypothesis was that this unusual combination is due to a fundamental abnormality in growth plate chondrogenesis.

Homozygous mutations in the aggrecan gene and aggrecan deficiency are associated with cartilage matrix deficiency in mice and nanomelia in chicks that exhibit severe dwarfism and premature death [6–8]. The growth plates in nanomelic chicks show abnormal morphology including higher cell density and reduced intercellular matrix. By evaluating the growth plates of nanomelic chicks at early stages of development until growth plate maturation, it was determined that nanomelic chondrocytes have initiation and progression through normal states of differentiation. However, at the time of growth plate maturation, the hypertrophic zone is small and disorganized. Nanomelic growth plates also show increased apoptosis in the proliferative zone and increased proliferation in the hypertrophic zone. In addition, the pre-hypertrophic and hypertrophic cells appear to overlap in the nanomelic mutant, suggesting the acceleration of hypertrophy and resultant precocious bone formation [7].

The first aggrecanopathy to be characterized was spondyloepimetaphyseal dysplasia, Kimberley type [9]. This autosomal dominant disorder encompasses short stature, stocky build, early onset osteoarthritis and radiographic changes including flattened vertebral bodies and flattened femoral epiphyses. Among the other aggrecanopathies, spondyloepimetaphyseal dysplasia, aggrecan type, is an autosomal recessive condition also caused by an aggrecan mutation with a phenotype of extreme short stature, macrocephaly, and radiographic changes [10]. Dominant familial osteochondritis dissecans is characterized by multiple osteochondritic lesions, disproportionate short stature, and early osteoarthritis. A heterozygous missense mutation in the aggrecan gene was found to cause this disorder [11].

Recently, whole exome sequencing has identified heterozygous defects in the aggrecan gene in 20 families leading to autosomal dominant short stature, bone age acceleration, and premature growth cessation. Multiple mutations have been found in these families, including 7 frameshift mutations and 8 truncating mutations [3–5]. Mutations were inherited in an autosomal dominant manner, with heterozygous mutations present in all affected family members and in no unaffected family members.

Little is known about how aggrecan deficiency affects cell signaling and proliferation leading to premature growth plate hypertrophy at the cellular level and accelerated growth plate maturation clinically. As aggrecan is a large extracellular matrix molecule, it is possible that the structural changes caused by aggrecan deficiency may be contributing to these growth plate changes. We hypothesized that aggrecan deficiency also affects cell signaling and proliferation, leading to premature chondrocyte hypertrophy. Using ATDC5 mouse chondroprogenitor cells, a well-characterized cell model of chondrogenesis, we sought to further characterize the effects of aggrecan deficiency on growth plate chondrogenesis.

## Materials and methods

### Cell culture conditions

ATDC5 mouse chondroprogenitor cells were purchased commercially (Sigma Aldrich, St. Louis, MO). ATDC5 mouse chondroprogenitor cells were used to conduct all experiments. For all experiments, cells were cultured in media consisting of a 1:1 mixture of Dulbecco's Modified Eagle Medium (DMEM) and Ham’s F12 containing 5% fetal bovine serum, 100 units/ml penicillin, 100 μg/ml streptomycin, 10 μg/ml human transferrin, and 3 x 10^−8^ sodium selenite, as previously described [12]. Puromycin 4 μg/ml was added to media for lentiviral-transduced cells. For experiments using reverse transcription quantitative polymerase chain reaction (qPCR), hemocytometer counting, and flow cytometry, cells were plated a density of 2.5 x10^4^ cells/well in a 12-well tissue culture treated polystyrene plate. When cells reached 50–70% confluence (after 3 days), insulin-transferrin-selenium (ITS) (Thermo Fisher Scientific, Franklin, MA), was added at a concentration of 10 μg/ml (Day 0) to stimulate chondrogenesis [12]. Media was changed every 2–3 days. Cells were studied on Days 0, 4, 8, 12, 16, and 21.

### Aggrecan knockdown

ATDC5 cells were transfected with aggrecan MISSION shRNA lentiviral transduction particles (SHCLNV-NM_007424 Sigma Aldrich, St. Louis, MO) to create aggrecan knockdown cell lines (AggKD). Non-target shRNA control transduction particles (SHC016V) were used to generate a control ATDC5 cell line. A puromycin kill curve determined that 4 μg/ml was the lowest concentration of puromycin required to kill cells that were not transduced. The lentiviral transduction was carried out as follows. Cells were plated at 2,000 cells per well in a 96-well plate. When cells reached 50–70% confluence, lentivirus transduction particles were added at multiplicity of infection of 5 along with polybrene at 8 μg/ml. After 24 hours, media was replaced with media containing puromycin 4 μg/ml. Media was replaced every 2–3 days, and cells were progressively transferred to larger plates after reaching near-confluence.

Cells were grown for 12 days with and without ITS to stimulate chondrogenesis. qPCR for aggrecan and Alcian blue staining were then used to assess the degree of aggrecan knockdown. The 2 lines with the greatest reduction in aggrecan were used for further experiments (AggKD 1 and AggKD 2 cell lines).

### Quantitative polymerase chain reaction

Cells were lysed and mRNA extracted using the RNAqueous total RNA isolation kit (Invitrogen, Carlsbad, CA), according to manufacturer. RNA was reverse transcribed into cDNA using iScript cDNA Synthesis Kit (Bio-Rad, Hercules, CA) according to the manufacturer. qPCR was done using the Bio-Rad CFX96 or Bio-Rad Connect machine. The housekeeping gene 18S was used for all qPCR data shown. Messenger RNA transcript levels were quantified using the delta delta Ct (ΔΔCt) method, normalized to rRNA 18S expression as follows: X = 2 ^-ΔΔCt^, in which ΔΔCt = (CtExp–Ct18S)–(CtCtl–Ct18S) and X = Relative transcript; CtCtl = Ct of control group. Beta-actin was also used as a housekeeping gene with results showing the same trends when this was used in place of 18S. Primer sequences for qPCR are provided in Table 1.

**Table 1:** Primer sequences used for quantitative PCR.

Gene	Species	Forward*	Reverse*	Accession Number	Amplicon Size
*Aggrecan*	Mouse	GCCTACCCGGTACCCTACAG (61.1°C)	ACATTGCTCCTGGTCTGCAA (59.9°C)	NM_007424.3	175
*18S*	Mouse/ Human	CGGCTACCACATCCAAGGAA (57.1°C)	GCTGGAATTACCGCGGCT (58.5°C)	NR_003278.3	187
*Beta-Actin*	Mouse	TGAGCTGCGTTTTACACCCT (59.9°C)	GCCTTCACCGTTCCAGTTTT (59.0°C)	NM_007393.5	198
*Col10a1*	Mouse	CTGCTGCTAATGTTCTTGAC (51.5°C)	ACTGGAATCCCTTTACTCTTT (51.1°C)	NM_009925.4	143
*Col2a1*	Mouse	ATCTTGCCGCATCTGTGTGT (57.3°C)	CTCCTTTCTGCCCCTTTGGC (58.8°C)	NM_031163.3	170
*Sox9*	Mouse	CAAGAACAAGCCACACGTCA (55.8°C)	TGTAATCGGGGTGGTCTTTC (54.5°C)	NM_011448.4	221
*Ihh*	Mouse	GCTCGTGCCTCTTGCCTACA (59.8°C)	CGTGTTCTCCTCGTCCTTGA (56.6°C)	NM_010544.3	163
*Col1a1*	Mouse	TTCTCCTGGCAAAGACGGAC (59.9°C)	CTCAAGGTCACGGTCACGAA (59.9°C)	NM_007742.4	247
*Col3a1*	Mouse	AAGGCTGCAAGATGGATGCT (60°C)	GTGCTTACGTGGGACAGTCA (59.9°C)	NM_009930.2	95
*Fabp4*	Mouse	AGCTGGTGGTGGAATGTGTT (59.8°C)	AATTTCCATCCAGGCCTCTTCC (60.4°C)	NM_024406.3	90
*Lpl*	Mouse	TGCCCGAGGTTTCCACAAAT (60.2°C)	CCAGCTGAAGTAGGAGTCGC (60.2°C)	NM_008509.2	113

*Annealing temperature in parentheses

### Alcian blue staining

Media was removed and Alcian blue 0.02% in water was added to each well. The cells were left in Alcian blue overnight, then washed with HCl x1 and with water x3. Cells were allowed to dry overnight. Guanidine HCl was used for Alcian blue extraction and absorbance measured using a Spectra Max M2 microplate reader.

### Pre-conditioning of cell culture plates

To determine the effects of growing AggKD cells in the presence of aggrecan, wells were pre-conditioned with control lentivirus transduced ATDC5 cells. Cells were plated at 100,000 cells/well in a 12-well plate and grown to confluence over 4 days, then media was supplemented with ITS to promote chondrogenesis and cells were maintained for an additional 7 days. Cells were lysed with hypotonic buffer solution (water with 0.5% Triton X-100) and rinsed 6 times with Hank’s buffered salt solution (HbSS) as previously described [13]. Control cells and AggKD cells were then plated in these pre-conditioned plates in the same conditions as described above. We demonstrated positive Alcian blue staining of wells after growing cells for 4 days in culture and lysing cells as described to show remaining aggrecan in the wells (S1 Fig).

### Co-culture of ATDC5 cells with aggrecan knockdown cells

To evaluate the effects of maintaining AggKD cells in the presence of aggrecan-producing cells, each AggKD cell line was grown together with ATDC5 cells that had not been transduced with lentivirus. Cells were grown at a total density of 2.5 x 10^4^ cells/well in a 12-well plate consistent with other experiments, but with a 1:1 combination of AggKD cells and unmanipulated ATDC5 cells. Cells were grown in media with ITS but without puromycin for 10 days. After 10 days of growth, puromycin 4 μg/ml was added to the media and cells were grown for an additional 10 days in puromycin-containing media. Media was changed every 2–3 days. The experiment was terminated after a total of 20 days.

### Determination of cell number

Cells were trypsinized, washed with phosphate-buffered saline (PBS), stained with trypan blue, then counted using a hemocytometer. Because cells began to form aggregates at day 12, making cell counts inaccurate, analyses were carried out for up to 8 days in culture.

### Flow cytometry

Cells were trypsinized and washed with PBS x1, then fixed with 66% ethanol for 2 hours. After fixation, cells were again washed with PBS x1 then stained with propidium iodide (Abcam, Cambridge, UK) by resuspending the cells in 200 μl propidium iodide and RNase staining solution, as per the manufacturer’s protocol. Flow cytometry was performed using an Accuri-C6 flow cytometer. Cell cycle analysis was performed using the ModFit program to determine the percentage of cells in each of the cell cycle phases, for each cell line at each time point (ModFit settings were manual mode, excluding aggregates and debris, with a trapezoid S-phase shape). A FITC pre-conjugated CD44 antibody (CD44-FITC, Miltenyi Biotech, San Diego, CA, USA) was used to stain and quantify CD44+ cells, according to the manufacturer’s instructions. Isotype control antibody Mouse IgG1-FITC (Miltenyi Biotech, San Diego, CA, USA) was used to control for non-specific binding of antibody to cells.

### Western blot

Control ATDC5 cells and AggKD 1 cells were plated for 12 days in culture in 6-well plates for the experiment. Total protein was extracted using 1X RIPA Buffer containing 1mM PMSF (Cell Signaling Technology, Danvers, MA, USA). Cells were washed with sterile cold PBS before the lysis. The lysis extract was collected and centrifuged at 12,000 RPM for 10 min at 4°C. The concentration of total protein supernatant was determined using Pierce BCA Protein Assay kit (ThermoFisher Scientific, Waltham, MA, USA). Then, 3X Blue Loading Buffer containing DTT, reducing agent (Cell Signaling Technology, Danvers, MA, USA) was diluted to 1X and mixed with the protein. The sample was boiled for 10 min at 95°C. Equal amounts of protein (100 μg) were resolved on 4–15% Mini-PROTEAN TGX Precast Gels (Bio-Rad, Hercules, CA, USA). Blots were transferred to PVDF membrane (Bio-Rad, Hercules, CA, USA) overnight at a constant voltage of 22V. Blocking of the membrane was done for 2 hour at room temperature with 5% Nonfat Dry Milk (Cell Signaling Technology, Danvers, MA, USA) in Tris Buffered Saline with Tween-20 (0.1%) (TBST). The membrane was incubated at 4°C overnight with specific primary antibodies at 1:1000 dilution, HAPLN1 (Cat. No.: A6616; ABclonal, Woburn, MA, USA) and β-Actin (Cat. No.: 8H10D10; Cell Signaling Technology, Danvers, MA, USA) and later incubated with secondary antibodies IRDye 680RD Goat anti-Rabbit and IRDye 800CW Goat anti-Mouse (LI-COR Biosciences, Lincoln, NE, USA) at 1:5000 dilution for 2 hours at room temperature. Membranes were imaged using an Odyssey fluorescence scanner (LI-COR Biosciences, Lincoln, NE, USA).

### Statistical analysis

Data are expressed throughout as mean and standard deviation. We used one-way analysis of variance (ANOVA) to determine significance of differences between the AggKD cell lines and the control line. A Tukey HSD post-hoc analysis was carried out when significance was p<0.05 (Vassar Stats). For experiments comparing no more than two experimental groups, a Student’s T-Test was used.

## Results

### Aggrecan knockdown in ATDC5 cells via lentiviral transduction of shRNA

**Fig 1:**
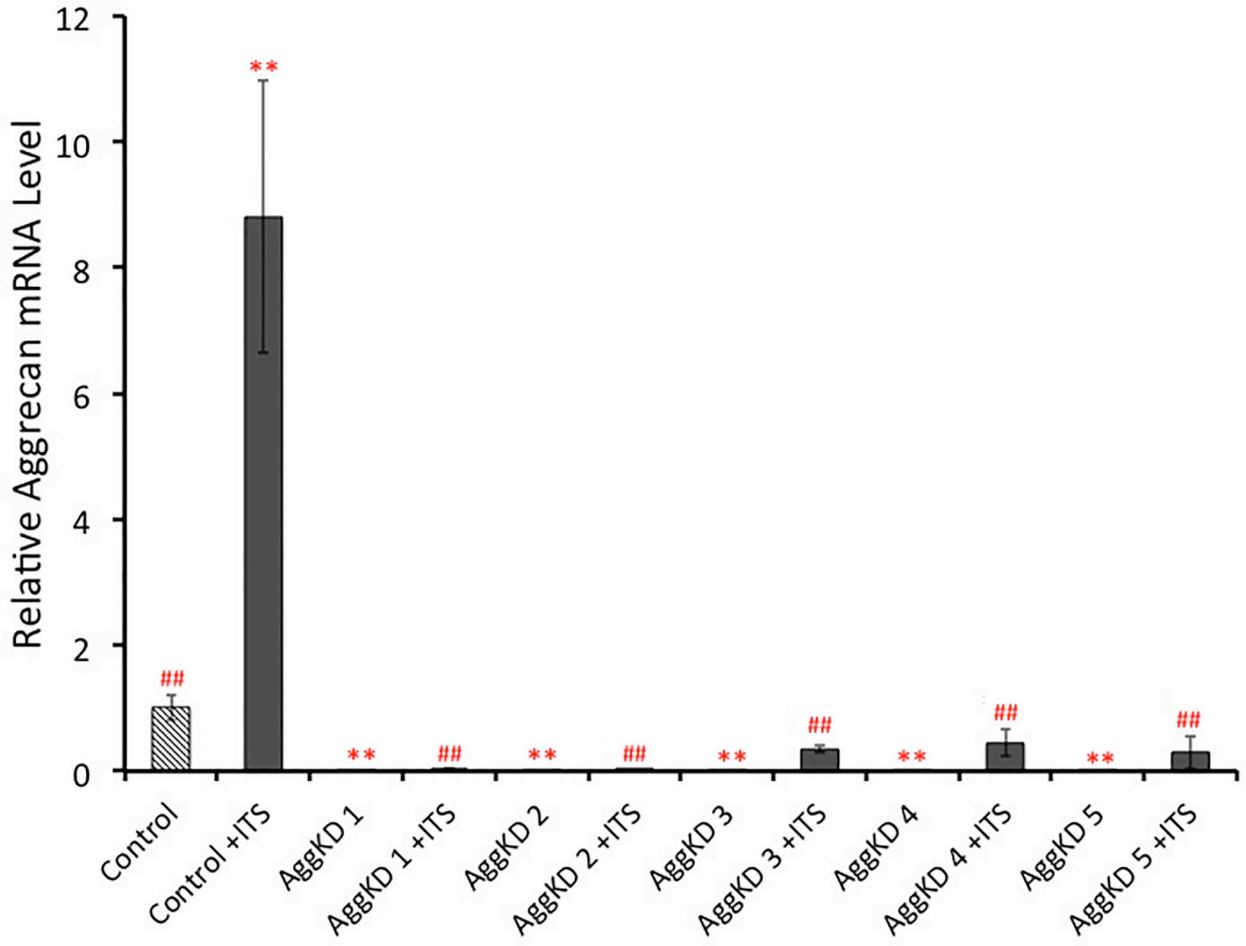
Relative Aggrecan mRNA expression levels, as measured by qPCR, in lentiviral-transduced control cells and 5 AggKD cell lines confirm decreased Aggrecan expression in the AggKD cells. Cells were cultured in media containing ITS for 12 days prior to being lysed for RNA preparation. Hatched bars represent unstimulated cells and solid bars represent cells stimulated with insulin to induce chondrogenesis. Data are shown as the mean ±1 SD for 4 biological replicates per group. ** p<0.01 versus control cells; ^##^ p<0.01 versus control cells +ITS.

**Fig 2:**
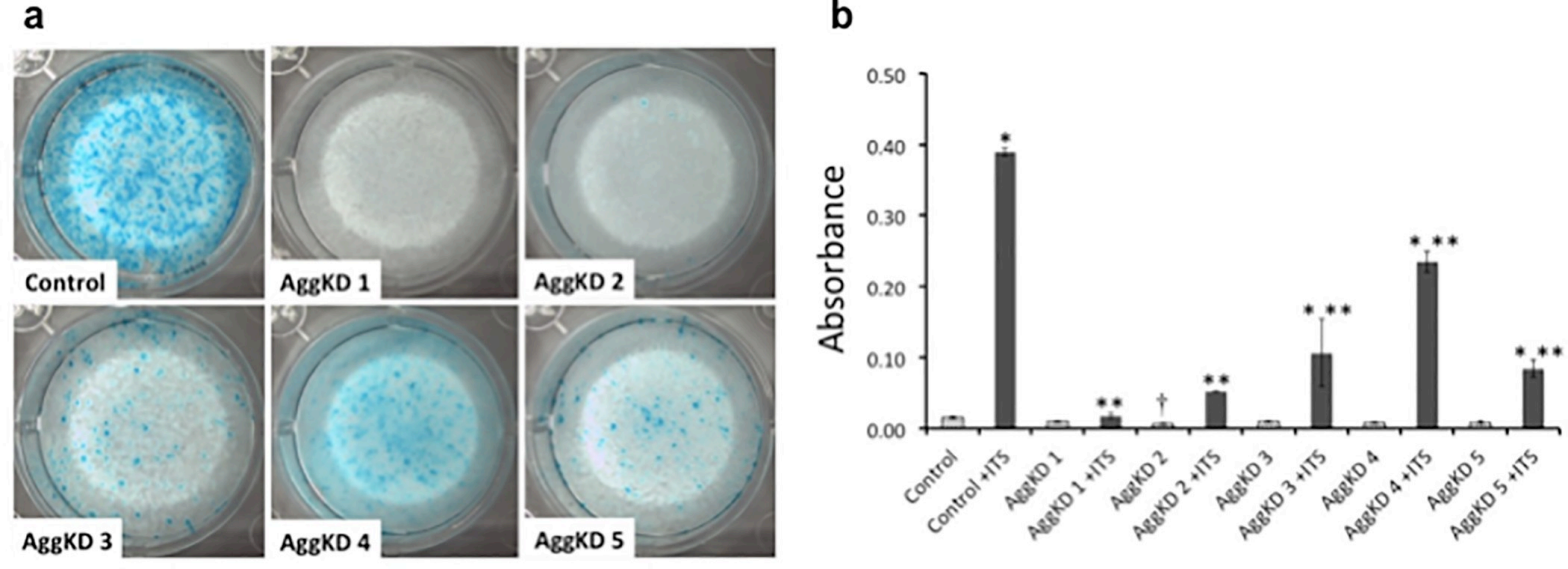
Aggrecan knockdown ATDC5 chondroprogenitor cells have decreased Alcian blue staining and absorbance levels compared to control cells after ITS stimulation. Cells were cultured in media containing ITS for 12 days at which they were fixed and stained with Alcian blue. (a) Representative stained cell culture plates are shown for each cell line. (b) Absorbance measurements after Alcian blue extraction are shown as the mean + 1 SD for 3 biological replicates per condition. Hatched bars represent unstimulated cells, and solid bars represent cells stimulated with ITS. *, p<0.01 versus control cells; ** p<0.01 versus control cells +ITS; †, p<0.05 versus control cells.

ATDC5 cells were transduced with lentiviral transduction particles to create AggKD cell lines and control cell lines. The level of aggrecan knockdown was assessed by qPCR (Fig 1) and Alcian blue staining (Fig 2). The control cell line exhibited higher levels of aggrecan mRNA expression when stimulated with ITS, compared to cells without ITS stimulation (p<0.01). Aggrecan mRNA expression levels in unstimulated and ITS-stimulated AggKD cell lines were comparable. All ITS-stimulated AggKD lines exhibited significantly less aggrecan expression than ITS-stimulated control cells (p<0.01).

We performed Alcian blue staining on all cell lines (Fig 2). Unstimulated control cells showed minimal staining. Unstimulated AggKD cell lines were similar to the unstimulated control cells except for AggKD 2, which showed even lower absorbance than the unstimulated control line (p<0.05). All ITS-stimulated AggKD cell lines had significantly less absorbance than the ITS-stimulated control cells. AggKD 1 and AggKD 2 cell lines had the lowest amounts of Alcian blue staining, consistent with the aggrecan qPCR results. These experimental cell lines, AggKD 1 and AggKD 2, were selected for further experiments as they exhibited the lowest levels of aggrecan expression.

### Markers of chondrogenesis in aggrecan knockdown cells vs. control cells

**Fig 3:**
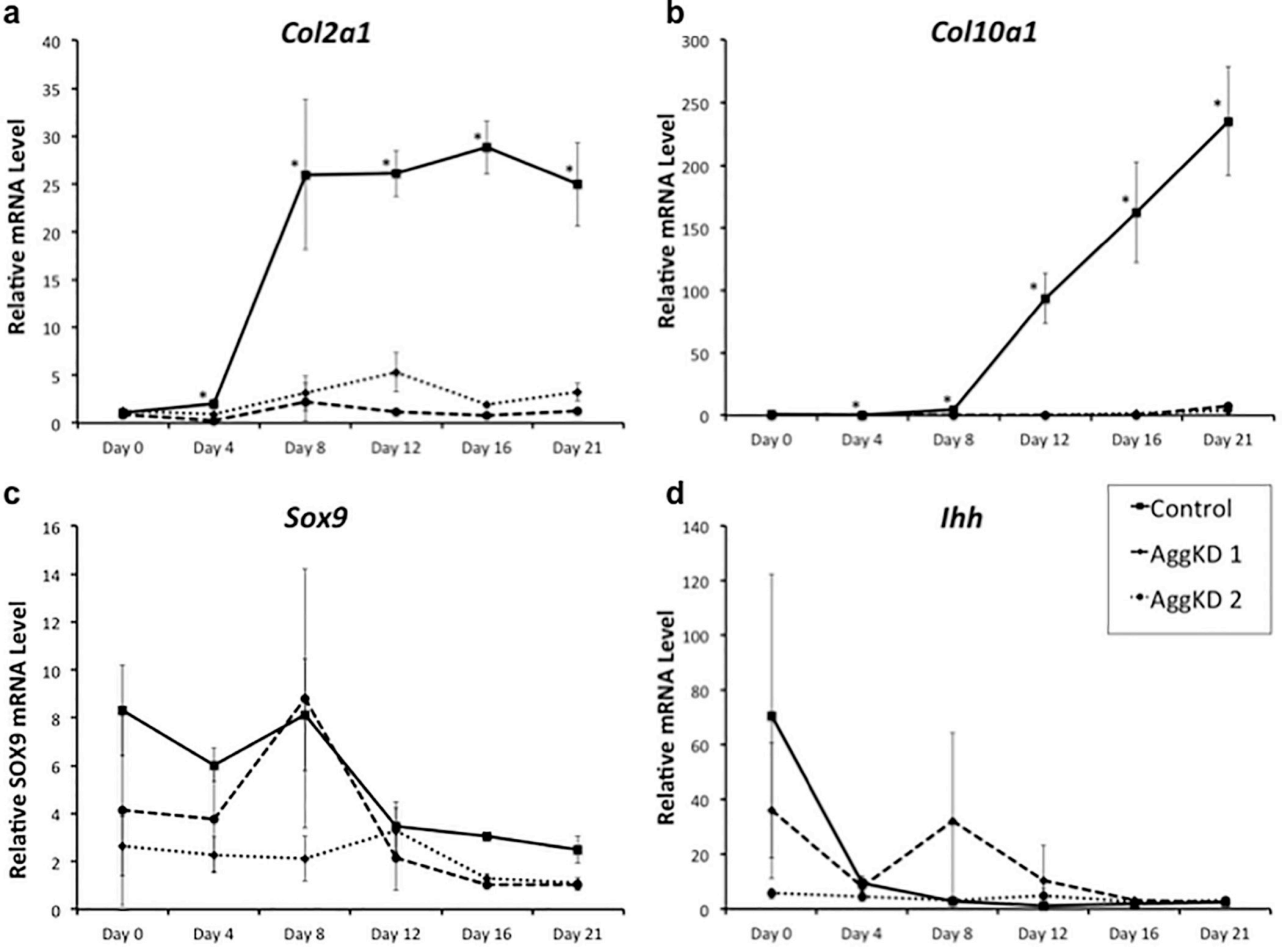
Chondrogenesis program is altered in Aggrecan knockdown ATDC5 chondroprogenitor cells. mRNA expression levels of *Col2a1* (a), *Col10a1* (b), *Sox9* (c) and *Ihh* (d) were determined by qPCR at day 0 through day 21 following chondrogenesis induction by the addition of ITS to culture media. Data for the control cells (solid line) and the two aggrecan knockdown cell lines, AggKD1 (dashed lines) and AggKD2 (dotted lines) are shown as the mean +1 SD for 4 biological replicates per condition. *, p<0.0001 versus AggKD lines.

We used qPCR for the quantification of type 2 collagen (*Col2a1*), type X collagen (*Col10a1*), SRY-Box 9 (*Sox9*), and Indian Hedgehog (*Ihh*) mRNA expression to evaluate the effect of aggrecan knockdown on chondrogenesis. These markers of chondrogenesis were evaluated at multiple time points in the control, AggKD 1, and AggKD 2 cell lines. Results show that control cells produced high levels of *Col2a1* by day 8, whereas AggKD 1 and AggKD 2 cell lines did not have increased *Col2a1* production through day 21 (Fig 3A). In control cells, *Col10a1* production greatly increased by day 12 and continued to increase through day 21. At no point did the AggKD 1 and AggKD 2 cell lines induce *Col10a1* expression (Fig 3B). Control lines had significantly higher *Col2a1* and *Col10a1* production starting from day 4, even prior to greater increases in mRNA levels in the control lines, through day 21 (p<0.0001). The expression levels of *Sox9* showed a trend of reduction in AggKD lines at all time points except Day 8 and Day 12 (Fig 3C). *Ihh* levels were similar between both AggKD cell lines and control cells (Fig 3D).

In order to determine whether aggrecan knockdown also affects molecules that are known to interact with aggrecan, such as hyaluronan receptor CD44 and Proteoglycan Link Protein (HAPLN1), we compared the expression of these molecules between control cells and AggKD cell lines. Flow cytometry analysis demonstrated that CD44 expression was unaltered by aggrecan knockdown (S2 Fig). Likewise, HAPLN1 protein production was also demonstrated to be unaltered as a result of aggrecan knockdown (S2 Fig).

Lastly, in order to determine whether aggrecan knockdown might cause ATDC5 cells to adopt a phenotype that is consistent with a non-chondrogenic cell lineage (i.e. fibroblastic phenotype, adipogenic phenotype), we compared expression of fibroblast/fibrochondroyte markers collagen 1 (*Col1a1*) and collagen 3 (*Col3a1*) as well as adipocyte markers Fatty Acid Binding Protein 4 (*Fabp4*) and Lipoprotein Lipase (*Lpl*) in control cells and AggKD cells (S3 Fig). AggKD cells exhibited lower *Col1a1* and *Fabp4* expression, compared to control cells. *Col3a1* and *Lpl* expression was unaffected.

**Fig 4:**
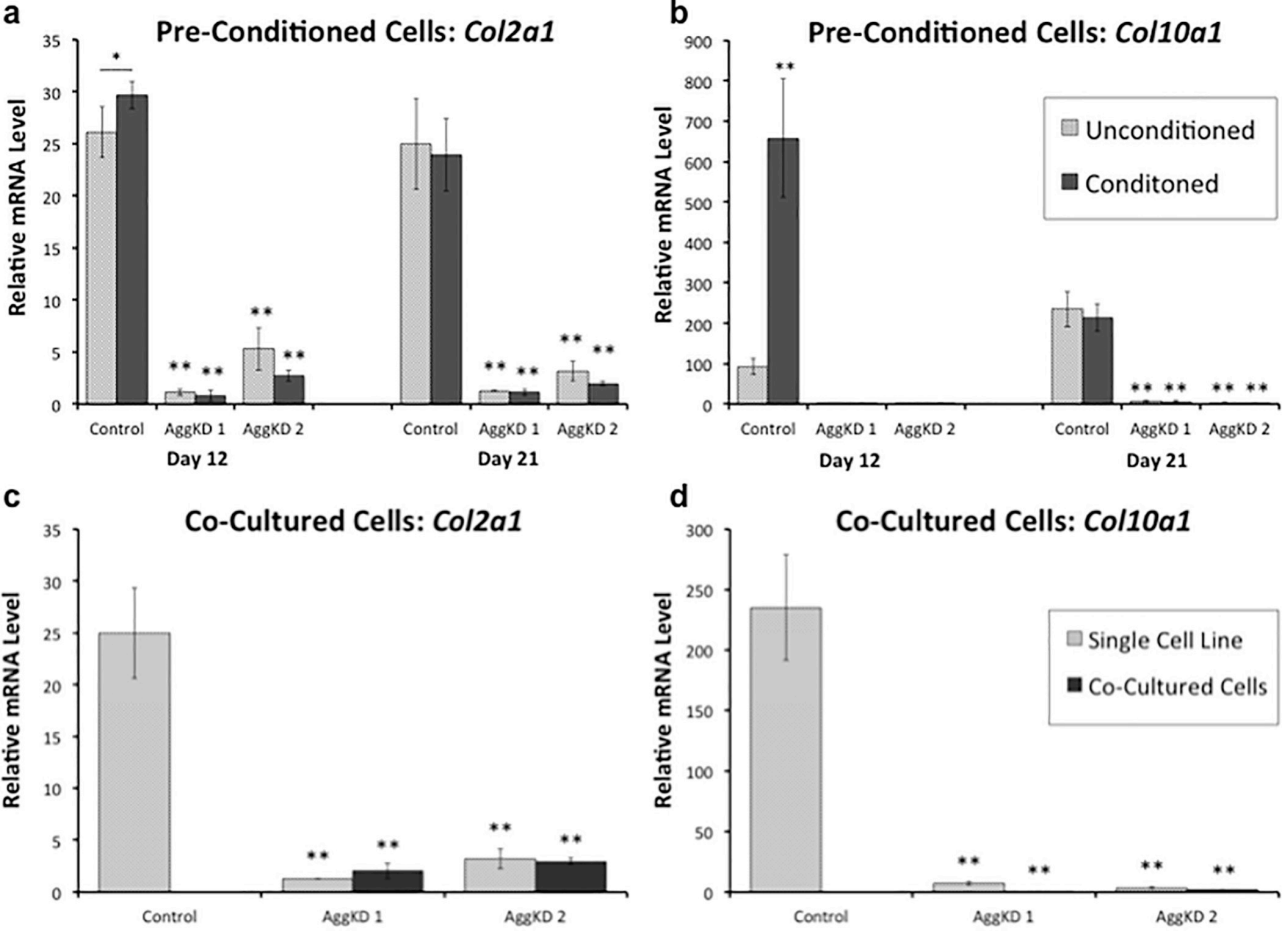
Aggrecan knockdown ATDC5 cells are unresponsive to extracellular matrix cues that normally accelerate the chondrogenic program. qPCR measurements to determine *Col2a1* (a) and *Col10a1* (b) expression were performed on RNA derived from lentiviral transduced control cells and 2 AggKD cell lines grown in cell culture plates that were untreated (hatched bars) or pre-conditioned with the extracellular matrix of control ATDC5 cells. qPCR measurements to determine *Col2a1* (c) and *Col10a1* (d) expression were performed on RNA derived from 2 AggKD cell lines grown individually (hatched bars) or co-cultured with wild-type ATDC5 cells. Before RNA extraction, wild-type ATDC5 cells were eliminated through puromycin selection. Data are shown as the mean +1 SD for 4 biological replicates per condition. *, p<0.05 versus corresponding unconditioned samples; **, p<0.01 versus corresponding unconditioned control samples.

In order to determine if the phenotype of the AggKD cells could be rescued by exogenous aggrecan, we cultured the control and AggKD cells in wells that were pre-conditioned by control ATDC5 cells grown in media containing ITS for 7 days after reaching confluence. At that point, the pre-conditioning cells were lysed, leaving behind only their extracellular and pericellular matrixes, and replaced with experimental cells. Based on Alcian Blue staining, we were confident that day 7 of cell growth was a sufficient amount of time for the establishment of extracellular matrix in the preconditioned wells. At day 12 of culture in pre-conditioned wells, control cells had increased levels of *Col2a1* and *Col10a1* expression compared to control cells grown in wells that had not been pre-conditioned (Fig 4A and 4B). There was no difference at day 21 in control cells grown in pre-conditioned versus unconditioned wells. Expression of *Col2a1* and *Col10a1* in the two AggKD lines was similar between pre-conditioned and unconditioned wells at both time points, and was significantly lower than expression in pre-conditioned control cells at both time points (p<0.01).

When AggKD cells were co-cultured in the same wells as standard ATDC5 cells, there was also no increase in *Col2a1* (Fig 4C) and *Col10a1* (Fig 4D) expression in AggKD cells. At day 20 of cell growth, the expression of *Col2a1* and *Col10a1* was similar between AggKD cell lines that had been co-cultured compared to those that had been cultured on their own. The control line was not co-cultured in this experiment.

### Effect of reduced aggrecan expression on proliferation and cell cycle kinetics

**Fig 5:**
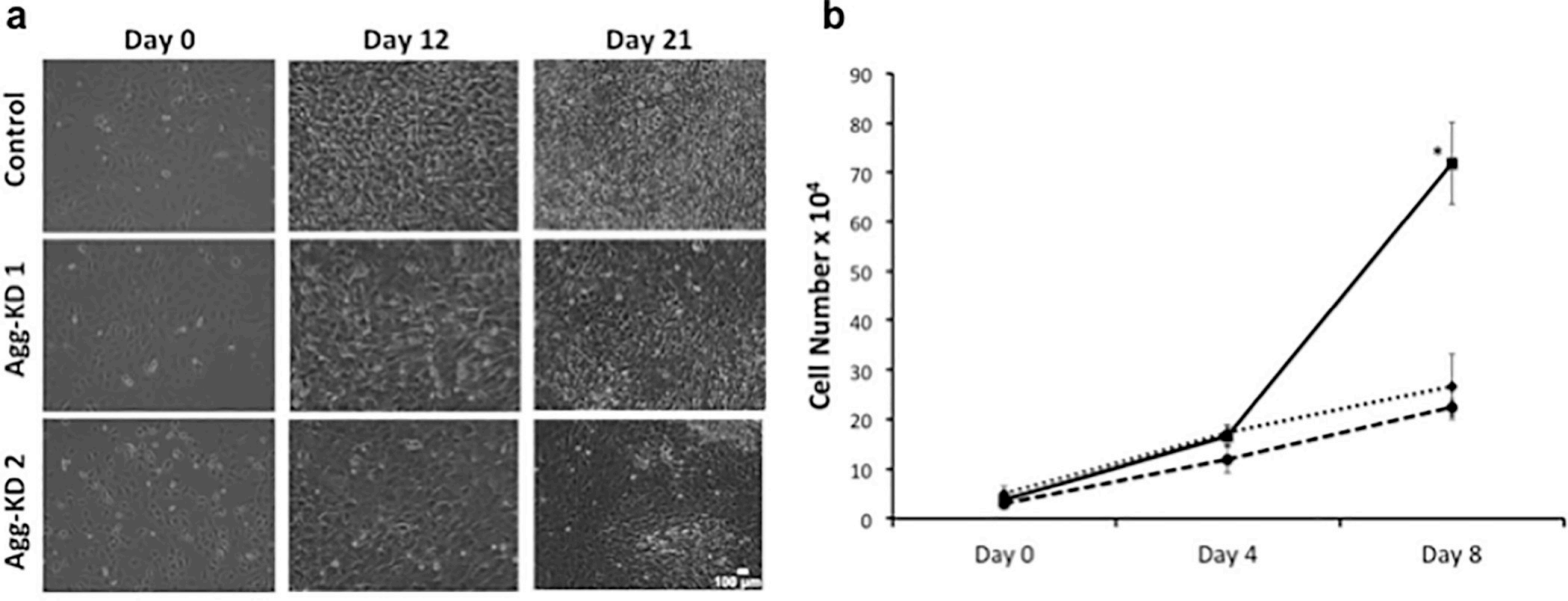
(a) Phase contrast photomicrographs of ITS-stimulated ATDC5 cells on days 0, 12 and 21 in culture. Images were acquired at 10x magnification. Scale bar in the bottom right corner depicts 100 micrometers. (b) Hemocytometer counts of ITS-stimulated controls cells (solid line) and aggrecan knockdown cells AggKD1 (dashed line) and AggKD2 (dotted line) on days 0, 4 and 8. Data are shown as the mean +1 SD for 3 biological replicates per condition. *, p<0.0005 versus both aggrecan knockdown cell lines.

**Fig 6:**
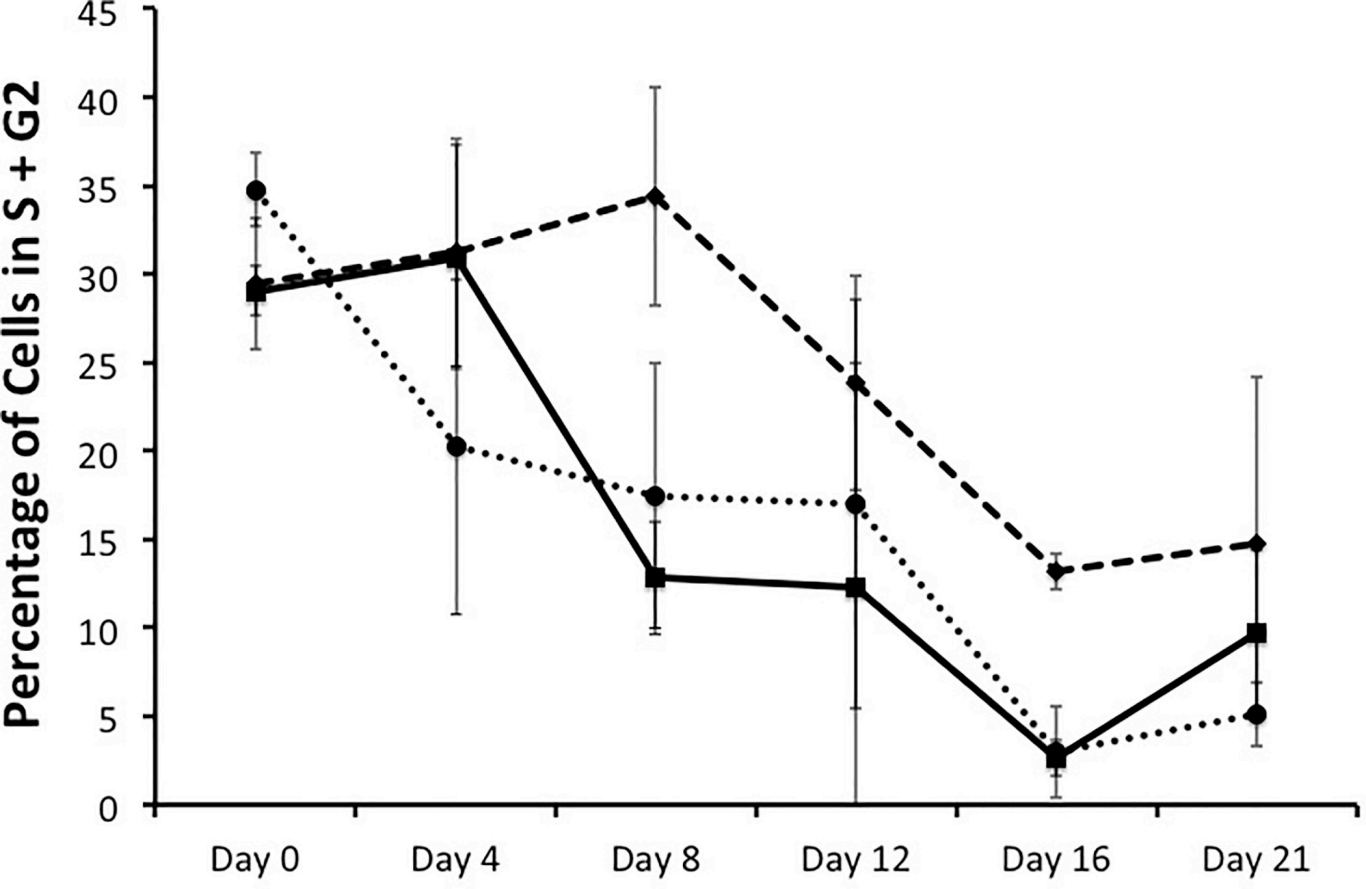
Proliferation is similar in control ATDC5 chondroprogenitor cells compared to aggrecan knockdown cells. Cells cultured in the presence of ITS were assessed by flow cytometry with propidium iodine staining. Data, representing the sum of cells in the S and G2 phases of the cell cycle are shown as the mean + 1 SD for 3 biological replicates per condition. Control cells, solid line; AggKD 1, dashed line; AggKD 2, dotted line.

Photomicrographs of ITS-stimulated control and aggrecan knockdown cells (Fig 5A) indicated greater cell density in the control cells at day 12 in culture. These results were confirmed by cell counting (Fig 5B). Flow cytometry with propidium iodide staining (S4 Fig) was used to assess differences in proliferation in each cell line by analyzing the percentage of cells in each phase of the cell cycle. The proportion of cells in S phase plus G2 phase (Fig 6) was similar in the control line and the two AggKD lines at all time points between day 0 and day 21.

## Discussion

The basis for the present studies was the unusual combination of impaired linear growth and advanced bone age that we observed in a kindred with a mutation in the gene encoding aggrecan [5]. This observation led us to hypothesize that intracellular aggrecan has an integral role in chondrogenesis. Our study supports this hypothesis. In particular, a marked reduction in aggrecan expression was associated with impaired induction of *Col2a1* and *Col10a1*, two extracellular matrix markers of chondrogenesis [14]. Expression of *Col2a1* is typically seen in the proliferative zone and the pre-hypertrophic zone of growth plates, whereas *Col2a1* is typically expressed in the hypertrophic zone. We therefore interpret our results as indicating that a marked reduction in aggrecan expression interrupts the early stages of chondrogenesis and prevents it from progressing normally.

Our results showed a marked reduction in *Col2a1* and *Col10a1* in AggKD cells (Fig 3A and 3B). This is in contrast to previous studies of animal models using nanomelic chicks and cartilage matrix-deficient mice which showed some expression of *Col2a1* and *Col10a1* in the growth plate, but in abnormal patterns [8,9]. Growth plates in nanomelic chick embryos showed abnormal morphology that included higher cell density and reduced intercellular matrix [7]. There was also disorganization of the growth plate with increased proliferation in the hypertrophic zone, increased apoptosis in the proliferative zone. By evaluating the growth plates of nanomelic chicks at early stages of development until growth plate maturation, these investigators determined that nanomelic chondrocytes have initiation and progression through normal states of differentiation, but at the time of growth plate maturation the hypertrophic zone is small and disorganized. In addition, the pre-hypertrophic and hypertrophic cells appear to overlap in the nanomelic mutant, suggesting the acceleration of hypertrophy and resultant precocious bone formation [7]. The observed difference in chondrogenic differentiation could be due to the fact that nanomelic chick and matrix-deficient mice still contain residual aggrecan mutant, while our AggKD cells exhibit a robust knockdown of the wildtype aggrecan gene. Thus, our *in vitro* loss-of-function study may have unexpectedly revealed the requirement of the aggrecan gene in chondrogenic differentiation. Our supplemental data also confirmed that aggrecan knockdown does not impact CD44 and HAPLN1, both of which are known to either interact with and/or form complexes with aggrecan protein. This suggests that neither of these molecules are involved in the attenuated chondrogenic phenotype we are reporting.

While our study provides information on the absence of aggrecan in the knockdown cells, this may have a different effect compared to a mutated aggrecan gene leading to a truncated protein or post-translational changes. In addition, many aggrecanopathies observed in humans are caused by autosomal dominant mutations in the aggrecan gene. This may allow some aggrecan to be produced, which may be sufficient for potential intracellular signaling effects despite reduced extracellular matrix.

Differences may also be due to additional factors contributing to chondrogenesis *in vivo* that may not be present in a cellular model, which relies on cell-autonomous effects. To test this hypothesis, we performed a rescue experiment using conditioned medium or extracellular matrix that contains wildtype aggrecan. AggKD cells grown in pre-conditioned wells had low expression of *Col2a1* and *Col10a1* showing that pre-conditioning wells did not rescue AggKD cells to undergo chondrogenesis (Fig 4A and 4B). Control cells grown in pre-conditioned wells had higher expression of *Col2a1* and *Col10a1* at day 12 of ITS exposure compared to cells grown in unconditioned wells. This suggests that the chondrogenesis program was accelerated when cells were grown in the presence of extracellular matrix. Co-culturing AggKD cells with unmanipulated ATDC5 cells was also unsuccessful at rescuing the deficiency in chondrogenesis (Fig 4C and 4D). What is more, our study shows that knocking down aggrecan has no effect on the synthesis of other cell surface and ECM protein molecules known to interact with aggrecan (i.e. CD44, Hapln1). Taken together, we interpret these results as suggesting that the role of aggrecan in chondrogenesis may involve an intracellular mechanism.

The results of our experiment measuring gene expression of markers and regulators of chondrogenesis showed similarities and also differences from some prior studies. In previous studies of ATDC5 cell differentiation with similar culture conditions, aggrecan gene expression begins at day 10 in culture, after *Col2a1* and before *Col10a1* is expressed [15]. This is consistent with our findings, however this time frame may vary in other experiments with different cell culture conditions. In all tested time points except Day 8 and Day 12, we found that there was a trend of reduced *Sox9* mRNA levels in AggKD ATDC5 cells, compared to control ATDC5 cells. While the differences did not meet a p-value 0.05, this trend of *Sox9* expression is in agreement with previous findings by Lauing et al [8]. *Sox9* is a transcription factor that regulates chondrogenic gene transcription at multiple points in chondrogenesis [16]. *Ihh* mRNA levels, on the other hand, were not different in AggKD cells compared to control ATDC5 cells. It is possible that our results vary to some extent from previous findings by Lauing et al. due to the fact that we are using an *in-vitro* chondroprogenitor model (not mature chondrocytes) in this study to look at the effects of aggrecan knockdown, whereas Lauing et al. used a transgenic mouse model to knockout aggrecan in *Col2* positive chondrocytes [8]. Additionally, basal *Sox9* and *Ihh* mRNA transcript levels are already low in ATDC5 mouse chondroprogenitors, as evidenced by late Cycle Threshold values (i.e. late 20s and early 30s) that we observed for these markers in our RT-PCR experiments. This may further explain why the reduction of these two markers could not be prominently detected in response to aggrecan knockdown.

We also observed that cell counts were significantly lower in both AggKD cell lines relative to control cells on day 8 of ITS-stimulation (Fig 5B). We did not detect differences in proliferation rates based on cell cycle analysis (Fig 6). In addition, the flow cytometry profiles of AggKD cell lines did not show an early peak of propidium iodide staining indicating apoptosis, thus making apoptosis an unlikely factor accounting for the lower AggKD cell numbers (S4 Fig). We considered the possibility that reduced aggrecan expression does not alter proliferation rate, but instead results in cells that are less adherent to the cell culture plates, resulting in low cell counts as live cells may have been removed when the plates were washed prior to counting. However, increased cell loss was not apparent via microscopy prior to media changes.

Consistent with these interpretations, growth plates in aggrecan-null mouse embryos also lacked matrix and chondrocyte organization and differentiation. Lauing et al. showed that these growth plates displayed abnormal patterns of *Col10a1*, *Sox9*, *Ihh*, *Ptch1*, and *Fgfr3* expression [8]. Comparison of the growth plates in aggrecan-null mouse embryos with rescue embryos expressing chick aggrecan showed partial reversal of the aggrecan-null phenotype. Taken together, these studies suggest that aggrecan has a significant role in the signaling pathways involved in chondrogenesis, and the lack of which leads to chondrodysplasia.

Limitations of this study include the use of a chondroprogenitor cell line rather than primary cells. While primary cells may provide a model that is more consistent with *in vivo* processes, the number of primary chondroprogenitor cells is extremely small in mice and hence difficult to isolate in reasonable quantities. Additionally, our current results do not elucidate the exact mechanisms of how aggrecan deficiency affects chondrogenesis. The failure of exogenous aggrecan to rescue the phenotype suggests that this may be an intracellular mechanism. However, further studies are necessary to confirm this hypothesis.

## Conclusions

Ours are the first *in vitro* studies suggesting that aggrecan is an essential molecule in cell-autonomous chondrogenesis. Further studies are needed to evaluate additional cell signaling molecules that may be affected by aggrecan deficiency to further study what may be an intracellular role in cell signaling.

## Supporting information

S1 Fig Alcian blue staining of preconditioned wells show aggrecan deposition in wells after growing for 4 days in culture and lysing cells as described in the “Pre-conditioning of cell culture plates” protocol. Wells were then stained with 0.02% Alcian blue solution, as described in our manuscript. Aggrecan knockdown ATDC5 cells are included as a control to demonstrate how the lack of aggrecan production impacts Alcian blue staining intensities.
(TIFF)

S2 Fig Flow cytometric analysis of hyaluronan receptor CD44 in (a) control ATDC5 cells, and Aggrecan knockdown ATDC5 cells. Empty peaks represent isotype control antibody staining. Red peaks represent CD44 antibody staining. (b) Mean percentage of cells that are CD44-positive amoung Aggrecan knockdown ATDC5 cells and control ATDC5 cells. Error bars represent ±1 SD of the mean. (c) Western blot analysis of Proteoglycan Link Protein levels in control ATDC5 cells, and Aggrecan knockdown ATDC5 cells. Actin is used as a loading control. Experiments were repeated three times.
(TIFF)

S3 Fig Mean relative mRNA expression levels of (a) Collagen 1, (b) Collagen 3, (c) Fatty Acid Binding Protein 4, and (d) Lipoprotein Lipase between Aggrecan knockdown ATDC5 cells and control ATDC5 cells. Error bars represent ±1 SD of the mean. *, p<0.05, **, p<0.01 versus control cell group.
(TIFF)

S4 Fig Representative flow cytometry analysis of samples from each designated cell line showing results of propidium iodide staining at days 0, 4 and 21. In all cases, cells were cultured in the presence of ITS.
(TIFF)

## Abbreviations

AggKD: aggrecan knockdown
Fabp4: Fatty Acid Binding Protein
qPCR: quantitative polymerase chain reaction
Lpl: Lipoprotein Lipase
ITS: insulin/transferrin/selenium
DMEM: Dulbecco's modified eagle medium
PBS: phosphate-buffered saline
Col2a1: type II collagen
Col10a1: type X collagen
Sox9: SRY-Box 9
Ihh: Indian Hedgehog
Col1a1: type I collagen
