# Supplementary figures and images for: Aggrecan is required for chondrocyte differentiation in ATDC5 chondroprogenitor cells
Source: PLoS One. 2019 Jun 17;14(6):e0218399. doi: 10.1371/journal.pone.0218399 (PMC6576788; doi:10.1371/journal.pone.0218399)

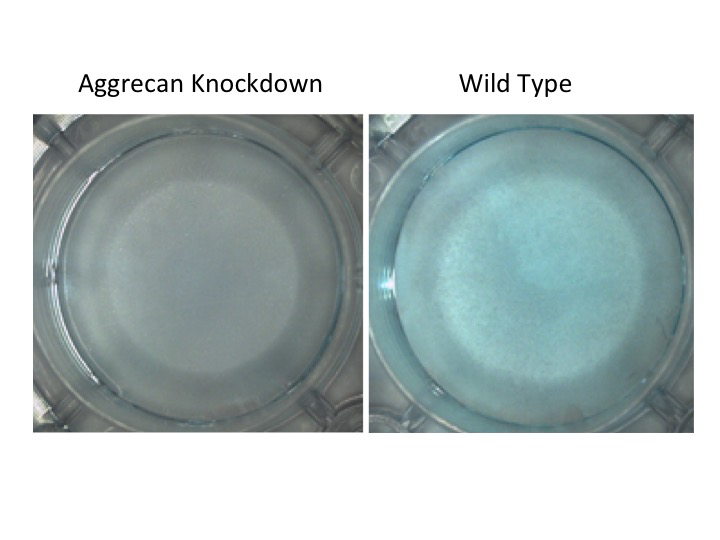

Supplement: S1 Fig — Wells were then stained with 0.02% Alcian blue solution, as described in our manuscript. Aggrecan knockdown ATDC5 cells are included as a control to demonstrate how the lack of aggrecan production impacts Alcian blue staining intensities. (TIFF) [file pone.0218399.s001.tiff]

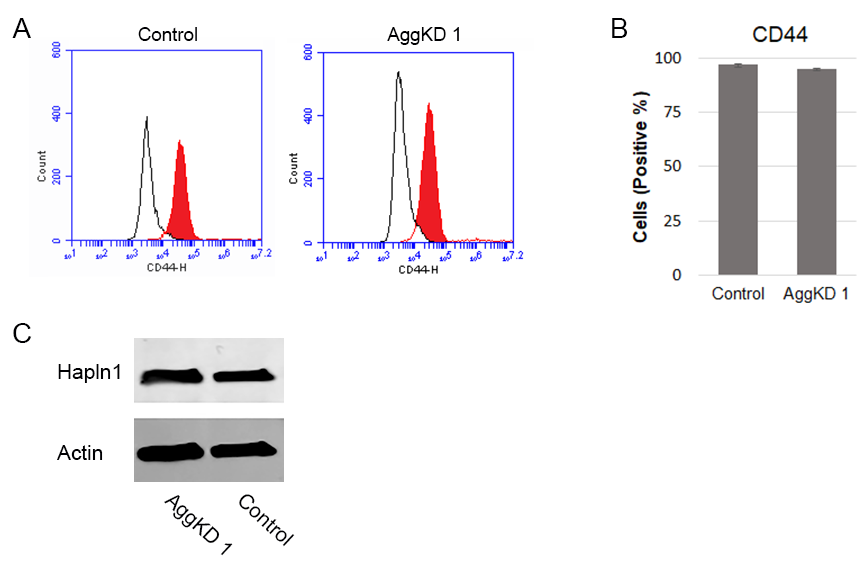

Supplement: S2 Fig — Flow cytometric analysis of hyaluronan receptor CD44 in (a) control ATDC5 cells, and Aggrecan knockdown ATDC5 cells. Empty peaks represent isotype control antibody staining. Red peaks represent CD44 antibody staining. (b) Mean percentage of cells that are CD44-positive amoung Aggrecan knockdown ATDC5 cells and control ATDC5 cells. Error bars represent ±1 SD of the mean. (c) Western blot analysis of Proteoglycan Link Protein levels in control ATDC5 cells, and Aggrecan knockdown ATDC5 cells. Actin is used as a loading control. Experiments were repeated three times. (TIFF) [file pone.0218399.s002.tiff]

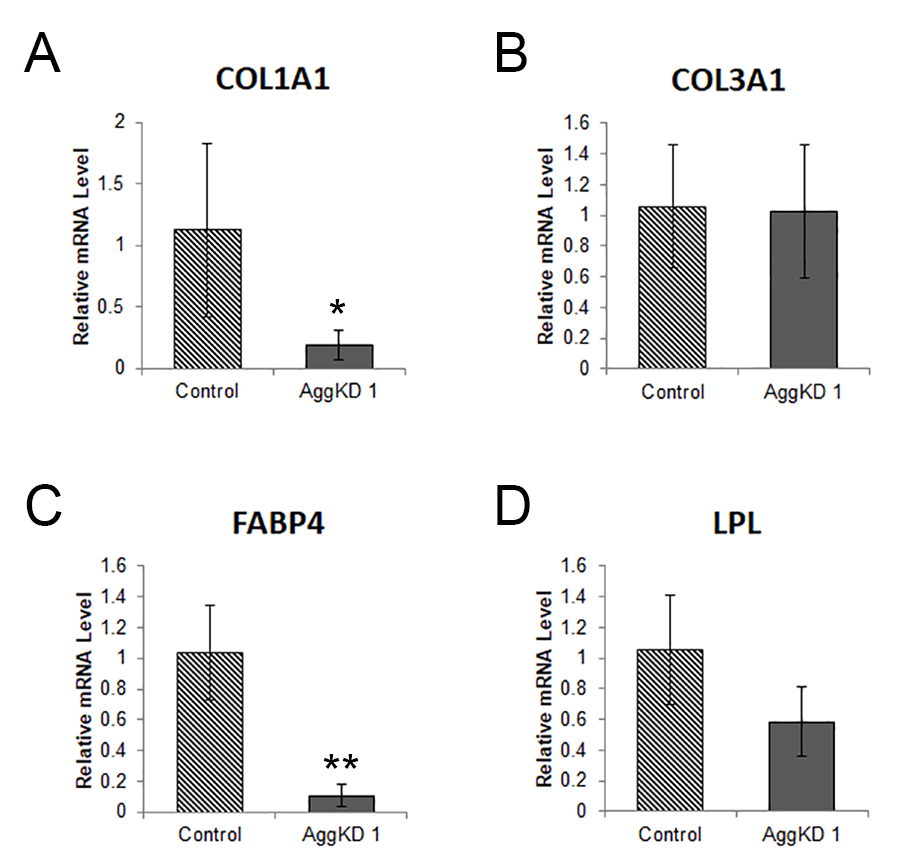

Supplement: S3 Fig — Mean relative mRNA expression levels of (a) Collagen 1, (b) Collagen 3, (c) Fatty Acid Binding Protein 4, and (d) Lipoprotein Lipase between Aggrecan knockdown ATDC5 cells and control ATDC5 cells. Error bars represent ±1 SD of the mean. *, p<0.05, **, p<0.01 versus control cell group. (TIFF) [file pone.0218399.s003.tiff]

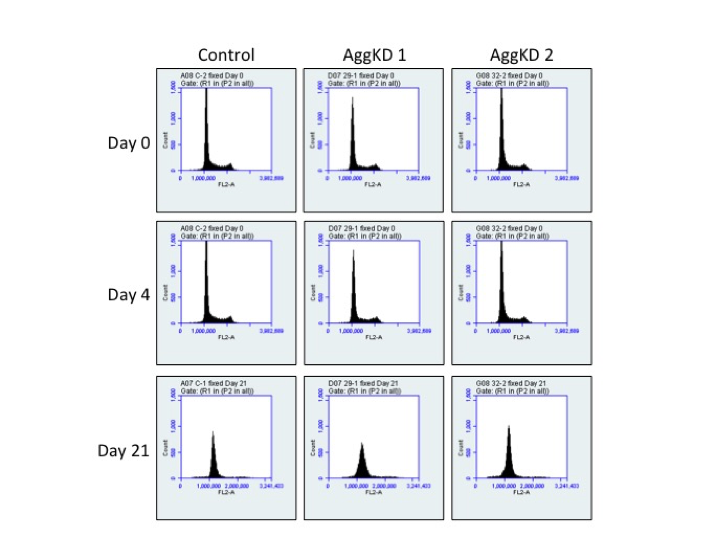

Supplement: S4 Fig — In all cases, cells were cultured in the presence of ITS. (TIFF) [file pone.0218399.s004.tiff]
